# Comparing manual and automated methods for calculating speaking rate in Parkinson's disease

**DOI:** 10.1121/10.0036021

**Published:** 2025-03-11

**Authors:** Lian J. Arzbecker, Kaila L. Stipancic, Jeremy D. W. Greenlee, Kris Tjaden

**Affiliations:** 1 Department of Communicative Disorders and Sciences, University at Buffalo, Buffalo, New York 14214, USA; 2 Department of Neurosurgery, University of Iowa, Iowa City, Iowa, 52242, USA; 3 Iowa Neuroscience Institute, University of Iowa, Iowa City, Iowa, 52242, USA lianarzb@buffalo.edu, klstip@buffalo.edu, jeremy-greenlee@uiowa.edu, tjaden@buffalo.edu

## Abstract

This study compared manual and automated methods for calculating speaking rate in recorded samples from individuals with Parkinson's disease. The manual procedure involved trained researchers measuring speaking rate through manual counting and acoustic analysis of speech units and pauses, while the automated method utilized a custom praat script developed by de Jong and Wempe [(2009). Behav. Res. Methods **41**(2), 385–390]. Results indicated moderate agreement between methods, strongest when the automated script was optimized per speaker. Despite the limitations of an automated approach, this research supports the potential of automation in speaking rate analysis and provides a basis for future refinement in clinical and research contexts.

## 1. Introduction

Speaking rate, defined as “the number of output units per unit of time” [Tsao *et al.* (2006), p. 1156], represents a fundamental aspect of spoken language production. Frequently expressed as a ratio of linguistic units, such as syllables or words, over time (measured in seconds or minutes), this metric encompasses multiple components of global timing. Speaking rate may be operationalized as having two components: articulation time and pause time. Together, articulation time and pause time are utilized to calculate speaking rate (Turner and Weismer, 1993; Jacewicz *et al.*, 2009). If there are no pauses, articulation rate equals speaking rate—but if pauses are present, articulation rate exceeds speaking rate. Pause time during connected speech can be influenced by several factors, including respiratory and cognitive capacities, among others (Huber *et al.*, 2012; Lofgren and Hinzen, 2022; Yunusova *et al.*, 2016). Thus, speaking rate provides a more comprehensive profile of global speech timing as compared to articulation rate.

### 1.1 Speaking rate in individuals with neurological conditions

Dysarthria, a motor speech disorder resulting from neurological damage or injury, often leads to reduced intelligibility (Darley *et al.*, 1969; Duffy, 2019). Intelligibility, the degree to which a listener comprehends a speaker's message (Munro and Derwing, 1995), is frequently reduced in individuals with neurodegenerative disorders, as dysarthria is a common symptom of these conditions (Yorkston, 2007). However, intelligibility alone does not capture the full impact of dysarthria. Yorkston and Beukelman (1981) observed that speaking rate differentiated speakers with dysarthria from a control speaker, despite comparable intelligibility ratings. Yunusova *et al.* (2016) analyzed oral passage readings from patients with amyotrophic lateral sclerosis (ALS), frontotemporal dementia (FTD), and healthy controls, and found that speaking rate had significant diagnostic value. Specifically, average speaking rates were significantly slower in three of the four ALS subgroups and all FTD subgroups compared to controls, with speaking rate effectively distinguishing between disorder subgroups. With an aim to increase intelligibility in speakers with dysarthria, rate control strategies—such as cued or metered speech, pacing boards, and delayed auditory feedback—have been used as therapeutic interventions for a variety of neurological diagnoses and dysarthrias including Parkinson's disease, cerebrovascular accident, and traumatic brain injury (Blanchet and Snyder, 2010; McAuliffe *et al.*, 2014; Van Nuffelen *et al.*, 2009; Yorkston *et al.*, 1990). Thus, speaking rate has clinical utility in diagnostic, prognostic, and treatment contexts.

### 1.2 Speaking rate calculation

Conventional methods for calculating speaking rate are often time-consuming and labor-intensive, typically involving manual transcription of audio recordings. Manual calculation first requires precise identification of temporal boundaries for speech and pauses, as informed by waveform and spectrogram data (Feenaughty *et al.*, 2013; McRae *et al.*, 2002). Manual calculation also relies on accurate syllable counts. The syllable itself can be challenging to define (Ladefoged and Johnson, 2011), and the same word may yield different syllable counts depending on the context and dialect in which the word is spoken. (Ernestus and Warner, 2011).

For example, Jacewicz *et al.* (2010) investigated speaking rate variation in American English dialects, examining both read sentences and spontaneous speech from male and female speakers. Results revealed both main effects and interaction effects across regional dialect, speaking task, and speaker sex. These findings, along with the variability in acoustic measurements of timing and the linguistic measurements of syllables—which can vary based on the investigator's interpretation—contribute to the overall complexity of manual methods. Consequently, there have been numerous attempts to develop automated methods for calculating speaking rate over the past few decades (Dekens *et al.*, 2007; Morgan *et al*., 1997; Narayanan and Wang, 2005). In contrast, with a few exceptions, studies of dysarthria continue to rely on manual measurement of speaking rate (Feenaughty *et al.*, 2021; Kuo *et al.*, 2014; Lam and Tjaden, 2016).

Green *et al.* (2004) compared manual and algorithmic methods for estimating speaking rate in individuals with dysarthria secondary to ALS and healthy controls. The algorithm of Green *et al.* (2004) was implemented in matlab using a custom, semi-automatic program called Speech Pause Analysis (SPA). Speech events in SPA were defined as stretches of continuous speech bounded by pauses. These events were delineated based on amplitude thresholds in the waveform: regions above the signal amplitude threshold were classified as speech, while regions below were classified as pauses. The program further processed these stretches by merging speech regions separated by pauses shorter than 200 ms and pauses separated by speech regions shorter than 50 ms. Thus, SPA featured three adjustable parameters: minimum pause and speech event durations, both measured in milliseconds, and minimum signal amplitude, expressed as a percentage. The algorithm was slightly less accurate with dysarthric speech, identifying more pause and speech events than manual annotations. This discrepancy may be due to the challenges of accurately detecting pauses and speech events in dysarthric speech, which can be more variable and less predictable than in typical speech. The authors noted that fine-tuning pause thresholds might be necessary, individualized by speaker. Manual calculation of speaking rate for the 60-word sample required approximately one and a half hours per speaker, whereas SPA completed the task in just 30 s.

Barnett *et al.* (2020) further investigated the psychometric properties of the “Bamboo Passage” using SPA by comparing the algorithm of Green *et al.* (2004) to another automated method of speaking rate calculation, the Speech Intelligibility Test (SIT) (Yorkston *et al.*, 2007). The SIT software performs various speech analysis tasks, including quantifying speaking rate in words per minute from a list of sentences. Barnett *et al.* (2020) examined speech from over 500 speakers, including individuals diagnosed with ALS and healthy controls, who provided recordings for both the “Bamboo Passage” and the SIT. Among nine speech and pause event variables, speaking rate was the main predictor of bulbar symptomatic status in the final logistic regression model. Comparable predictive utility was observed for both the “Bamboo Passage” and the SIT.

Additional automated techniques have been developed by de Jong and Wempe (2009) and de Jong *et al.* (2021). Over the years, the authors have investigated the use of syllable nuclei detection as a way of automatically measuring speaking rate. The technique involves comparing relative intensity peaks to the surrounding intensity. This method will be discussed in greater detail in Sec. 2.4, as this particular method is the focus of the current study. The de Jong script was of interest for several reasons. The script is publicly available, offers flexibility with adjustable parameters, and the latest version was specifically updated to detect filled pauses—an important future goal for analyzing conversational speech samples without a transcript. Ultimately, the current study aimed to determine whether an automated method of calculating speaking rate produces results comparable to those obtained through manual procedures, as this metric can clinically quantify disease progression in dysarthria associated with neurological conditions.

The aim of this research was to compare automated and manual methods of calculating speaking rates. This comparison sought to determine whether an automated approach can produce results that are both as accurate and reliable as those obtained through traditional manual calculations. More specifically, this study evaluated the effectiveness of the algorithm of de Jong *et al.* (2021)—which was trained on typical L1 Dutch and L2 English—when applied to a novel sample: L1 English of speakers with Parkinson's disease. Thus, we assessed whether the algorithm provides reliable estimates of speaking rate and pause patterns in a clinical population characterized by known variability, despite the script not being tested on this group.

## 2. Methods

### 2.1 Participants

A total of 60 speakers from a larger, ongoing longitudinal study provided speech samples for this study. Participants ranged in age from 47 to 77 years (M = 60.4, SD = 10.2) and had been diagnosed by a movement disorder neurologist with idiopathic Parkinson's disease (PD) without atypical Parkinsonism features. All participants had elected to undergo bilateral implantation of deep brain stimulation (DBS) electrodes in the subthalamic nucleus.

Additional inclusion criteria included proficiency in English as a primary language and no requirement for hearing aids. PD severity was assessed by a trained examiner using the Movement Disorder Society-Unified Parkinson's Disease Rating Scale Part III (MDS-UPDRS) (Goetz *et al.*, 2008). Speech severity was documented using the score from the speech question of the MDS-UPDRS Part III as well as percent correct words transcribed from the sentences from the SIT, as judged by three untrained listeners (Yorkston *et al.*, 2007). Listeners independently transcribed the SIT sentences presented via REDCap in a quiet room while wearing headphones. Prior to judging each test, listeners heard an additional SIT sentence produced by the speaker that was not part of the transcription task and were instructed to adjust the volume of the headphones to a comfortable listening level. Listeners were instructed to write what they heard word-for-word. Listener transcriptions were scored using Autoscore (Borrie *et al.*, 2019; Stipancic *et al.*, 2024). The percentage of words correctly transcribed were averaged for the three listeners to yield an overall percent correct score for each test. See Appendix A1 in the supplementary material for speaker demographics and characteristics.

### 2.2 Stimuli and recording procedures

The stimuli consisted of 15 unique sentence lists, each list composed of 18 sentences selected from the SIT (Yorkston *et al.*, 2007). The SIT is widely used in clinical research and utilizes a pool of 1100 unique sentences sourced from *Reader's Digest* articles. The linguistic characteristics of a given sentence vary greatly [see Stipancic *et al.* (2023) for a detailed lexical analysis]. For the current study, each set of 18 sentences was manually curated from the SIT pool to ensure phonetic diversity. This approach addressed limitations of the SIT software, which generates semi-random lists that may not always be phonetically balanced. Orthographically, each of the 18 sentences within a given list ranged from five to 12 words (M = 8.50; SD = 1.68) and five to 25 syllables (M = 12.24; SD = 3.45) in length. On average, each list contained a total of 153 words (SD = 2.84) and 220 syllables (SD = 10.07).

Because the speakers were sourced from an ongoing project, not all participants had completed the entire protocol at the time of this study's analysis. The protocol included recording sentence lists at three timepoints: presurgical baseline, six months post-surgery, and 12 months post-surgery—with the latter two time points featuring recordings with DBS both on and off. In this study, 60 unique speakers contributed a total of 120 sentence sets, with 20 speakers contributing one set, 20 speakers contributing two sets, and 20 speakers contributing 3 sets. Eight different speakers contributed to each of the 15 lists, resulting in a total of 2160 sentences for analysis (calculated as 60 speakers × 1–3 sentence lists per speaker × 18 sentences per list), which equates to 120 discrete sentence list recordings. These samples represented contributions across various time points and DBS conditions. Participants read the sentences from a computer monitor while wearing a Countryman E6 omnidirectional non-occluding earset microphone connected to a Focusrite Scarlett Solo preamplifier. The audio was recorded at a sampling rate of 22.05 kHz using praat (Boersma and Weenink, 2022).

### 2.3 Manual speaking rate calculation

TF32 (Time-Frequency Analysis Software Program for 32-bit Windows) (Milenkovic, 2011) was used for acoustic segmentation. Trained researchers utilized dual waveform-wideband (300–400 Hz) spectrogram displays while listening to the audio to segment sentences into runs and pauses. A run was defined as a stretch of uninterrupted speech without pauses, while a pause was defined as a nonspeech interval (silent or filled) with a duration of at least 0.20 s (Goldman-Eisler, 1968; Turner and Weismer, 1993). Standard acoustic criteria (e.g., stop release bursts, frication, or voicing bars) were used to determine the beginning and end of each run (McRae *et al.*, 2002; Tjaden and Wilding, 2004). Syllable counts were guided by orthographic transcriptions of each sentence, with counts based on the actual speech produced, accounting for reduced forms in connected speech and idiolect variation (e.g., *finally* counted as two or three syllables depending on production).

From this manual procedure, the following measures were calculated for each sentence: number of syllables, run duration, total sentence duration, articulation rate, and speaking rate. Note that only number of syllables and total sentence duration (used to calculate speaking rate) were relevant to the current study. Run duration (pauses excluded) and total sentence duration (pauses included) were measured in seconds. Articulation rate was calculated by dividing the number of syllables by run duration, and speaking rate was derived by dividing the number of syllables by total sentence duration. Articulation rate was calculated on a run-by-run basis (with a single sentence potentially containing multiple runs), while speaking rate was calculated on a sentence-by-sentence basis and averaged across all 18 sentences to obtain a single, average measure of speaking rate for each sentence set for use in the statistical analysis. Approximately 20% of the data were remeasured to assess both intra- and inter-rater reliability. Pearson correlation coefficients were 0.99 and 0.98, respectively, indicating excellent reliability both within and across raters.

### 2.4 Automated speaking rate calculation

The run segments identified in the manual procedure required reconstruction before automated processing. To prepare each sentence list, run segments were concatenated in r, version 4.3.1 (R Core Team, 2023) using the “sound” package (Heymann, 2023). No additional preprocessing occurred prior to supplying the concatenated audio files to praat.

The automated method selected for the current study was an adapted version of the publicly available praat script by de Jong *et al.* (2021), which utilized syllable detection from relative intensity peaks surrounded by intensity dips. This script was an update to the custom script of de Jong and Wempe (2009), which calculated silent periods and speaking rate. The adaptations in the current study preserved the core functionality of the script, with modifications primarily focused on streamlining data management (e.g., adding a CSV file-saving function). The steps are summarized below, but readers are encouraged to consult de Jong *et al.* (2021) for a more detailed description.

The script identifies syllable nuclei using intensity and voicing criteria. First, intensity is used to identify peaks in the energy profile, detecting potential vowel segments assumed to exhibit greater energy within the syllable. Subsequently, the algorithm ensures these peaks are sufficiently spaced apart to avoid multiple detections within a single syllable. Voicing analysis is then applied to exclude peaks corresponding to voiceless consonants that may erroneously register as high intensity. See Appendix A2 in the supplementary material for an example of the script's output.

**Fig. 1.**
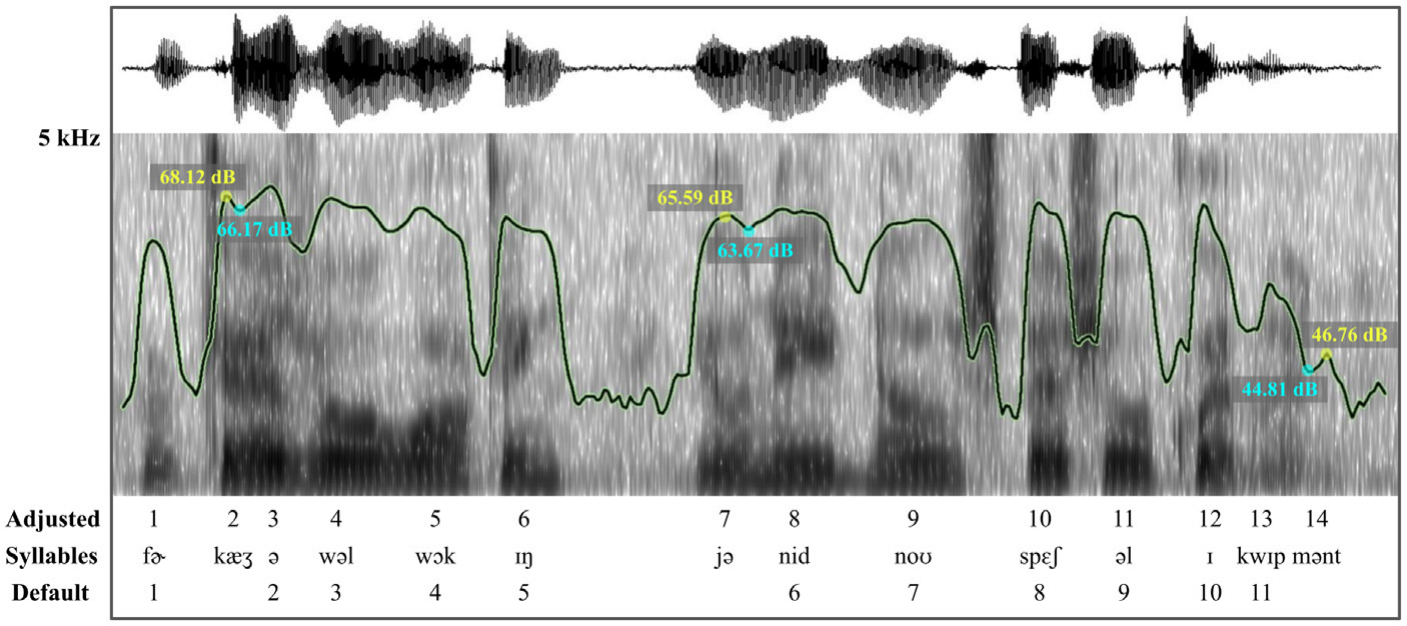
Intensity contour (highlighted in green) superimposed on a spectrogram for the sentence, “For casual walking, you need no special equipment.” Both adjusted and default syllable counts and a broad phonetic transcription are shown. Using the default peak dip of 2 dB yielded a syllable count of 11; however achieving an accurate syllable count of 14 required adjusting the dip level to 1.15 dB. Note that the intensity difference between each yellow peak and blue dip is less than 2 dB, causing the script to disregard these dips under default parameters.

When running the script, a dialogue box prompts users to review settings before proceeding. The default values for syllable nuclei detection autofill, but the script allows customization of three parameters: silence threshold, minimum dip near peak, and minimum pause duration. The silence threshold determines the intensity difference a section must have relative to the median intensity of the entire sound file. The minimum dip near peak defines the intensity decrease that surrounding dips must have relative to the potential syllable peak. Minimum pause duration is the shortest duration labeled as silence. This degree of customization lends itself to numerous applications; however, for the current experiment, only the minimum dip near peak was systematically customized. The silence threshold was not adjusted because visual comparison of the TextGrid along with the waveform and spectrogram confirmed that background noise was sufficiently differentiated from speech. The minimum pause duration was uniformly set to 0.20 s to align with previous studies and was not adjusted (Tjaden and Wilding, 2004; Turner and Weismer, 1993).

According to de Jong and Wempe (2009), a minimum preceding dip of 2 dB is recommended for unfiltered sound and 4 dB for filtered sound. Given that the speech samples in the current study were not filtered, initial testing adhered to the default minimum peak value of 2 dB. However, the script consistently and substantially underestimated the number of syllables produced by the speaker, which suggested that the high-fidelity audio warranted a more sensitive dip parameter. To balance precision with efficiency, iterations of the script were run at 30 different dip levels, initially in 0.05 dB increments from 0.05 to 1.30 dB, and then in larger increments (1.5, 1.75, 2, 2.25 dB) as changes in the output became minimal beyond 1.25 dB. This approach avoided the impracticality of evaluating an infinite number of minute increments between zero and two, while still providing a thorough examination of potential settings.

Running one iteration of the 120 sentence lists took approximately one minute. Since speakers read relatively short sentences from a screen, the script's filled pause detection features were not utilized. The results from all iterations were compiled into a comprehensive dataset. A user-designed function was employed to select the optimal automated speaking rate and identify its corresponding minimum peak dip value for each of the 120 sentence lists. The criterion for selecting the “optimal” script measurement was the degree of correlation with the corresponding manual measurement (i.e., the gold standard). Figure 1 compares optimized and default syllable counts.

### 2.5 Statistical analysis and modeling

All statistics were computed in r (R Core Team, 2023). Pearson correlation coefficients were calculated to assess the relationship between manual and automated speaking rate methods. Specifically, correlations were calculated for the automated speaking rate measured under default parameters and the manual speaking rate. An additional correlation was performed between the manual speaking rate and the absolute difference between the manual and automated speaking rates to evaluate the consistency of the automated method. To compare the strength of the correlations obtained from the default and optimized automated methods, Fisher's *Z*-transformation was applied. Additionally, correlations between manual and automated speaking rates and dysarthria severity were computed to assess how each measure relates to overall severity. These analyses aimed to evaluate whether the automatic measure could serve as a valid substitute for the human-based measure.

To evaluate multiple sources of variability, a generalizability (G) study was conducted using the “gtheory” r package (Moore, 2016). G theory extends classical test theory, including the intraclass correlation coefficient (ICC), by allowing for the simultaneous evaluation of several sources of variability [Cronbach *et al.* (1963); see Xue *et al.* (2023) for speech-specific applications]. Based on the G study results, mixed effects modeling was conducted. The initial model was expressed as Eq. (1),

$$\text{speec}h_{\text{rate}}∼\;\left(1|\text{speaker}\right)+\left(1|\text{recording}\right)+\left(1|\text{time}\right)+\left(1|\text{DBS}\right)+\left(1|\text{list}\right)+\left(1|\text{method}\right),$$ (1)

where *speaker* = individual speaker (60 levels), *recording* = individual sentence list recording (120 levels), *time* = timepoint (3 levels), *DBS* = DBS on/off (2 levels), *list* = unique sentence list (15 levels), and *method* = measurement method (2 levels).

It is important to note that timepoint and DBS condition were originally modeled as random intercepts in Eq. (1), but the small number of levels for these factors—three for timepoint and two for DBS condition—could have yielded inaccurate estimates. Thus, two separate fixed-effects models were performed to determine whether timepoint or DBS condition had a significant effect on speaking rate. The first model indicated that timepoint did not have a significant effect on speaking rate (6-month: *p* = 0.059; 12-month: *p* = 0.315; *R*^2^ = 0.030). Similarly, the second model suggested that DBS condition was not a significant predictor of speaking rate (on: *p* = 0.849; off: *p* = 0.122; *R*^2^ = 0.025). Once it was confirmed that timepoint and DBS condition contributed minimally to the total variance, a simplified model was adopted to increase interpretability, as expressed in Eq. (2),

$$\text{speec}h_{\text{rate}}∼\;\left(1|\text{speaker}\right)+\left(1|\text{recording}\right)+\left(1|\text{list}\right).$$ (2)

## 3. Results

The correlation between the manual and automated speaking rates was moderately positive (*r* = 0.772, *p* < 0.001). For 119 of the 120 sentence lists, the automated method consistently underestimated speaking rate compared to manual calculations. The manual speaking rate and the absolute difference between the manual and automated speaking rate exhibited a strong positive correlation (*r* = 0.887, *p* < 0.001), with faster (higher) manual speaking rates associated with lower accuracy by the automated procedure. Similar results were observed for articulation rate. The correlation between manual and automated articulation rates was moderately positive (*r* = 0.804, *p* < 0.001).

However, after selecting the optimal (i.e., most strongly correlated with the manual measurement) adjusted parameter for each of the 120 lists individually, a correlation of 0.954 (*p* < 0.001) was achieved. Fisher's *Z*-transformation test revealed a significant difference between the correlations of the manual speaking rate with the default and adjusted automated methods (*Z* = 6.48, *p* < 0.001), indicating a statistically significant improvement in performance with the optimized settings. This optimization process involved fine-tuning the parameters for each list based on the correlation with human annotations, which may have led to an inherent bias toward improving accuracy. See Appendix A3 in the supplementary material for a visualization of the difference between running the script with default vs adjusted parameters. The limitations of this particular optimization technique begin to emerge around a manual speaking rate of approximately five syllables per second, as evidenced by the diminishing adherence to the dashed line representing perfect correlation. Similar results were observed for articulation rate. When the same optimal parameter for speaking rate was used, the correlation between manual and automated articulation rates increased significantly (*r* = 0.924, *p* < 0.001).

Further analyses examined the relationship between manual and automated speaking rates and dysarthria severity (as indexed by MDS-UPDRS Part III speech scores). No significant associations were found, likely because the speakers were only mildly impaired. While both measures of speaking rate showed weak positive correlations with severity, neither reliably represented severity in this dataset.

The simplified model informed by the G study was used to estimate variance components. See Appendix A4 in the supplementary material for the variance component summary. The G coefficient of 0.84 indicates a high level of reliability, with the majority of variance in speaking rate being attributable to differences between speakers rather than error or other factors. Specifically, the “Speaker” source accounted for 83.5% of the variance, highlighting the substantial contribution of individual speaker differences. The rate calculation method, whether automatic or manual, contributed minimally to the overall variance, suggesting that both methods are roughly equivalent in their measurement. The remaining variance was almost entirely attributable to individual list recordings and unique sentence lists, with the residual variance being relatively small (6.3%), further reinforcing the robustness of both measurement approaches.

## 4. Discussion

The findings of this study provide insights into the accuracy of an automated speaking rate calculation relative to the traditional manual method. Although the automated procedure approximates manual measures—as supported by the results of the G analysis—it consistently underestimates speaking rate when default parameters are applied. Since speaking rate is calculated by dividing the syllable count by the total duration of the audio file—and the same audio file was used for both the automated and manual methods (i.e., the duration remained constant)—the underestimation indicated that the automated method missed syllables, as illustrated in the supplementary material. Visual inspection of audio files and corresponding TextGrids revealed that the script tended to miss monosyllabic words at sentence onset or offset (e.g., “She was really shook up”) as well as unstressed syllables in function words and multisyllabic words (e.g., “We gathered shells on the beach”). Unidentified syllables were largely remediated by decreasing the minimum peak dip, suggesting that the intensity dips between certain syllables were insufficient for the script to identify. This issue was particularly noticeable in syllables with lower intensity, such as those at the edges of sentences or in unstressed positions.

The G study further demonstrated that the majority of variance in speaking rate is attributable to differences between speakers rather than methodological variability. Summary statistics of the tested peak dip thresholds (ranging from 0.05 to 2.25 dB) revealed an average optimal threshold of 0.30 dB (SD = 0.36). The variability in these thresholds suggests differences in how dip values impact performance across speakers. A critical next step is to explore why the automated method performs well for some speakers but not others. To begin this exploration, a linear mixed-effects model was used, which revealed that neither timepoint (0, 6, and 12 months) nor DBS on/off conditions had a significant impact on speaking rate. This suggests that the observed speaker-specific differences in algorithm performance are not attributable to these factors. Nonetheless, after revisiting the study aim, the following conclusions were drawn:

(1) The automated method provided a moderately strong correlation with manual speaking rate calculations (*r* = 0.772), but consistently underestimated speaking rate under default settings. However, by optimizing the parameters for each speaker, the correlation improved significantly (*r* = 0.954), indicating that the automated method can indeed approximate the accuracy and reliability of manual methods when tailored to individual speakers.
(2) The effectiveness of the algorithm of de Jong *et al.* (2021) in a clinical population with Parkinson's disease was mixed. The script calculated a reasonable estimate of speaking rate, but tended to miss syllables, particularly at sentence boundaries and in unstressed syllables. These results indicate that while the algorithm offers a foundation for automated speech analysis in clinical populations, further refinements are needed to ensure accuracy across diverse speech profiles.

Results have several practical implications for researchers and clinicians using automated speech analysis tools. Although these resources may offer significant time savings and consistency compared to manual methods, parameter adjustments are essential for optimizing accuracy of automated methods. Careful calibration of automated scripts should be regarded as a necessary step in the analysis process. Moreover, results highlight the potential limitations of using default parameters in commercially or freely available speech analysis tools. Here, the unmodified automated method consistently underestimated speaking rate compared to manual measures, with an average underestimation of 1.2 syllables per second. This stresses the importance of flexibility in adjusting parameters to better align with individual speaker characteristics and improve measurement accuracy. The speech used in this study was not severely degraded—based on the MDS-UPDRS speech score and intelligibility on the SIT (see the supplementary material)—suggesting that such parameter adjustments may be necessary even for speakers with only mild speech impairment.

### 4.1 Limitations and future directions

While this study shows the potential for using automated methods to calculate speaking rate in PD, there are limitations. First, the optimized method demonstrated significant improvement over the default settings, but the optimization process was time-consuming and required multiple iterations for each sentence set. Additionally, the identification of optimal settings was only made possible by referencing manual speaking rate measures. In practice, the automated method could potentially replace the need for manual remeasurement in assessing intra- and inter-rater reliability. Furthermore, the study focused on relatively short, read sentences, which may not fully reflect the complexities of spontaneous or conversational speech. Inclusion of an age- and sex-matched group of speakers free from neurological disease also would further advance understanding of how the automated method performs with normal speech. Future research could explore automating the parameter optimization process, reducing the need for manual adjustment and making the automated method more accessible for broader use. Examining the performance of the automated method across different speech contexts, such as spontaneous speech or speakers with varying speech pathologies, would further contribute to understanding the method's applicability in diverse settings. Finally, integrating machine learning techniques to adaptively adjust parameters based on speech characteristics may offer a promising direction for enhancing the accuracy and efficiency of automated speech analysis.

## Supplementary Material

See the supplementary material for speaker demographics and characteristics, for an example of the script's output, and for a visualization of the difference between running the script with default vs adjusted parameters.

## Data Availability

The data that support the findings of this study are available from the corresponding author upon reasonable request.
